# Recent Progress in Metal–Organic Framework-Derived Nanostructures in the Removal of Volatile Organic Compounds

**DOI:** 10.3390/molecules26164948

**Published:** 2021-08-16

**Authors:** Deval Prasad Bhattarai, Bishweshwar Pant, Jiwan Acharya, Mira Park, Gunendra Prasad Ojha

**Affiliations:** 1 Department of Chemistry, Amrit Campus, Tribhuvan University, Kathmandu 44618, Nepal; devalprasadbhattarai@gmail.com; 2 Carbon Composite Energy Nanomaterials Research Center, Woosuk University, 443 Samnye-ro, Samnye-eup, Wanju-gun, Jeonju-si 55338, Korea; bisup@woosuk.ac.kr (B.P.); acharyajione@woosuk.ac.kr (J.A.); 3 Woosuk Institute of Smart Convergence Life Care (WSCLC), Woosuk University, 443 Samnye-ro, Samnye-eup, Wanju-gun, Jeonju-si 55338, Korea; 4 Department of Fire Disaster Prevention, Woosuk University, 443 Samnye-ro, Samnye-eup, Wanju-gun, Jeonju-si 55338, Korea

**Keywords:** VOCs, MOFs, nanomaterials

## Abstract

Air is the most crucial and life-supporting input from nature to the living beings of the planet. The composition and quality of air significantly affects human health, either directly or indirectly. The presence of some industrially released gases, small particles of anthropogenic origin, and the deviation from the normal composition of air from the natural condition causes air pollution. Volatile organic compounds (VOCs) are common contaminants found as indoor as well as outdoor pollutants. Such pollutants represent acute or chronic health hazards to the human physiological system. In the environment, such polluted gases may cause chemical or photochemical smog, leading to detrimental effects such as acid rain, global warming, and environmental pollution through different routes. Ultimately, this will propagate into the food web and affect the ecosystem. In this context, the efficient removal of volatile organic compounds (VOCs) from the environment remains a major threat globally, yet satisfactory strategies and auxiliary materials are far from being in place. Metal–organic frameworks (MOFs) are known as an advanced class of porous coordination polymers, a smart material constructed from the covalently bonded and highly ordered arrangements of metal nodes and polyfunctional organic linkers with an organic–inorganic hybrid nature, high porosities and surface areas, abundant metal/organic species, large pore volumes, and elegant tunability of structures and compositions, making them ideal candidates for the removal of unwanted VOCs from air. This review summarizes the fundamentals of MOFs and VOCs with recent research progress on MOF-derived nanostructures/porous materials and their composites for the efficient removal of VOCs in the air, the remaining challenges, and some prospective for future efforts.

## 1. Introduction to the Metal–Organic Framework

Metal–organic frameworks are inorganic–organic porous crystalline materials with extremely high surface areas and significant chemical diversity. Metal ions or their clusters coordinated with organic ligands or linkers forming a one-, two-, or three-dimensional porous or void structure constitute a metal–organic framework (MOF) [1,2,3]. They are also called porous coordination polymers (PCPs) [4]. Ions of metal such as cobalt, copper, cadmium, zirconium, and iron, etc. can act as the central metal ion while organic moieties or compounds with carboxylates (e.g., benzene-1,3,5-tricarboxylate, 1,4-benzenedicarboxylate, etc.) or nitrogen-bearing compounds (e.g., bipyridines, imidazoles, azoles, etc.) can behave as the ligand (electron pair donor) or linker in MOF [5,6,7]. The bonding between metal ions (nodes) and linker could be oxygen coordinated or nitrogen coordinated. Various types of metal–organic frameworks are devised for different applications. For instance, Pang et al. developed “Quasi-Ce-MOF” as an electrocatalyst for the urea oxidation reaction [8]. In another work, Pang’s team synthesized nitrogen-doped hexagonal NiCoO nanoplates from Ni-Co-MOF for the application in electrochemical energy storage [9]. Besides the fabrication of nanomaterials, MOFs have been exploited as a sacrificial precursor for the synthesis of carbon nanomaterials, metallic compounds, and their composites with a tunable and controllable nanoarchitecture for various applications [10]. Besides the aforementioned applications, MOF-based materials are also being used for catalysis [11].

In MOFs, the metal ions act like nodes to bind the arms of the organic ligand to form a repeating cage-like structure with a profound internal surface area (1000–10,000 m^2^/g). Therefore, it can be regarded as a composition of metal nodes and organic linkers [12]. The effect of the cavity and pore size distribution (a few nanometers to several angstrom), functional groups, hydrophilic–hydrophobic properties and surface unsaturation impart significant applications of MOFs in various fields including the adsorption of gas molecules, volatile organic compounds, the catalytic degradation of various gaseous pollutants, etc. [13]. MOFs are structurally different from other traditional inorganic porous materials such as zeolite, mesoporous carbon (2–50 nm), and silica in terms of uniform pore structures, atomic level, structural uniformity, tunable porosity, extensive varieties and flexibility in network topology, dimension, and chemical functionality. MOF can be regarded as a synergistic feature of structure and compositions enhancing the high surface-to-volume ratio, low density, higher loading capacity and micro-reactor environment. Compared to inorganic microporous material such as zeolites, controlled architecture and pore functionalization impart flexible rational design for MOFs. In contrast to zeolite, MOFs differ in their construction having bridging organic ligands intact throughout the synthesis. The coordination number of metal ions dictates the number of binding ligands and their orientation influencing the shape, size, and orientation or pores. MOFs are different from the covalent organic framework (COFs) in the sense that the COFs are composed of light elements such as hydrogen, boron, carbon, nitrogen, and oxygen with extended structures.

Metal–organic frameworks have attracted much attention due to their versatile applications in the synthesis of porous nanomaterials, carbon materials, etc. applicable in various fields of research and technology such as supercapacitors, batteries and fuel cells, electro-catalytic reactions, water treatment, drug delivery, anti-angiogenesis and photodynamic therapy, catalysis, adsorption, removal of volatile organic compounds, oxygen reduction reaction, hydrogen evolution reaction, etc., and other possible fields [14,15,16,17,18,19,20,21,22]. Furthermore, open metal–organic frameworks have been widely used in separation chemistry, gas storage, and molecular recognition [23].

With the booming industrial development and urbanization, the level of air pollution has been alarmingly increasing over the world, inducing serious hazards to human health and the environment. The emission of a diverse level of pollutants such as nitrogen oxides (NOx), sulfur oxides (SOx), and carbon oxides (COx), volatile organic compounds (VOCs) into the environment can pose serious health problems and environmental implications as well as social ramifications. Among a list of airborne pollutants, VOCs are a class of remarkable pollutants [24,25,26]. Metal–organic frameworks have been used in the efficient removal of volatile organic compounds. The higher surface area, large pore volume, and specific gas adsorption potency of MOFs make them triumph over other common adsorbents for the removal of VOCs [27]. Post-fabrication changes of MOF can induce selective adsorption properties. MOFs can be executed for the synthesis of nanomaterials, which can be used for the removal of VOCs. Therefore, the synthesis of MOFs is crucial in the field of synthetic chemistry, separation chemistry, and the sensing of volatile organic compounds [28]. Metal–organic frameworks can be synthesized by any of the following methods:

- Solvothermal or hydrothermal: in this process, crystals are allowed to grow smoothly over the course of hours to days from a hot solution.
- Microwave-assisted solvothermal synthesis: in this method, microwaves can be used to nucleate MOF crystals rapidly from a solution.
- Chemical vapor deposition method: this is a solvent-free method for the synthesis of MOFs. In this method, initially, metal oxide precursor layers are deposited, followed by exposing to sublimed ligand molecules, which induces a phase transformation to the MOF crystal lattice.

Post-synthetic modification of MOFs:

Post-synthetic modification of MOFs helps to introduce some new sites with new chemical properties. It helps to increase the functionality of MOFs by reacting with metal–organic complexes with linkers. In this method, either the ligand or the metal ions are exchanged in pre-fabricated MOFs with a new ligand or metal ions by the exchange method [29]. The exchange is performed to tailor some specific functions onto the MOFs. For this purpose, the previously developed MOFs are washed with a solvent followed by soaking in a solution of new ligand or in a solution of new metal. The ligand exchange can be assisted by heating.

## 2. Volatile Organic Compounds

Volatile organic compounds are natural or synthetic, low-boiling organic compounds with smaller molecular mass and high vapor pressure existing at room temperature. VOCs may be polar or non-polar, aliphatic or aromatic, indoor- or outdoor-prevalent pollutants [30]. Naturally, VOCs are produced by plants, microorganisms, and animals. A large number of microbes can produce VOCs as a secondary metabolite during their growth, which are termed microbial VOCs [31,32]. Natural VOCs have their essential significance of protecting plants from stress, attracting insects for pollination and seed dispersal. Some VOCs, such as trans-anethole, estragole, eugenol, isoeugenol, camphor, thujones, etc., are the constituents of essential oil with therapeutic application [33,34]. Besides these, VOCs are produced from petrochemical industries, gasoline vehicles, solvent uses, dye and paint industries, building materials, cleaning products, and many synthetic compounds. VOCs may be aliphatic hydrocarbons or aromatic hydrocarbons and their derivatives. Usually, such VOCs are obtained as by-products from industry, agriculture, transportation, and day-to-day activities in households which potentiate either to vaporize or dissolve in water. Partially burnt fuels such as gasoline, diesel, petrol, etc. produces VOCs. Besides this, the uses of solvents, paints, wax, and some sorts of cosmetic products also produce VOCs [35,36,37].

### 2.1. Sources and Effect of VOCs

Many of the harmful VOCs are derived from anthropogenic activities such as the burning of fuels, leakage of harmful gases, industrial sewage discharge, e-waste, etc. [38]. Some of the common sources of harmful VOCs include formaldehyde, BTEX (benzene, toluene, ethylbenzene, and xylene), PAH (polycyclic aromatic hydrocarbon), styrene, tetrachloroethylene (used in dry cleaning), ethylene glycol, methylene chloride (used as paint stripper), 1,3-butadiene, 1,8-cineole, vinyl chloride, acetone, carbon tetrachloride, isopropylbenzene, undecane, etc. Combustion products of woods, fuels, diesel, gasolines, automobile emissions, and tobacco smoke also consist of VOCs. VOCs form a constituent in various commercial and household products such as many petroleum-based products, fumigants, moth repellents, carpets, paints, lacquers, varnishes, glues, perfumes, nail polishes, tobacco smokes, adhesives, dyes, rubber, plastics, and cleaners used in industries and manufacturing companies. VOCs are also present in personal care products such as perfumes, deodorants, lotions, and some pharmaceutical products [39,40].

Semi-volatile organic compounds (SVOCs) have relatively higher boiling points than VOCs and evaporate at a slow rate but can accumulate over a time. Some examples of semi-volatile organic compounds include chlorinated tris, fire retardants (PCBs, PBB), pesticides (DDT, chlordane), etc. In the global emission estimates, the major contribution of VOCs is biogenic sources [41].

Above a permissible level, VOCs can have acute or chronic effects on humans. Inhalation of VOCs in humans provides prompt absorption across the lungs, gastrointestinal tract, and skin. Short-term exposure to such chemicals is associated with headaches, irritability, depression, dizziness, allergies, asthma, difficulty with concentration, and irritation of delicate organs such as the skin, ears, eyes, nose, throat, etc. Long-term exposure to VOCs is likely to affect vital organs such as the liver, kidneys, nervous system, etc. Some VOCs can cause cancer to humans, even at low concentration. The extreme effect of such VOCs may lead to genetic disorders [42].

VOCs not only have an affect on humans but also equally affect the environment. Though VOCs tend to escape from the groundwater by evaporation, once they are dissolved in groundwater, they are more persistent. Furthermore, some VOCs are degraded by aquatic bacteria; still, some other VOCs are non-degradable and ultimately enter the food web or ecosystem. VOCs can form ground-level ozone or chemical smog and secondary organic aerosols (SOAs) by reacting with nitrogen oxides, which ultimately causes a detrimental effect on the environment. VOCs are one of the major sources of atmospheric photochemical reactions causing various environmental hazards [43].

### 2.2. Classification of VOCs

Not all compounds are equally volatile. The volatile compounds that evaporate faster are more hazardous and cause a more serious risk than others There is no clear demarcation for the categorization of volatile compounds, but the United States Environmental Protection Agency (EPA) has adopted World Health Organization (WHO) Guidelines to divide indoor organic pollutants into the following types:

A. Very Volatile Organic Compounds (VVOCs):

The boiling point of these compounds fall in the range of 0 to 50–100 °C. Some common examples are propane, butane, methyl chloride, etc.

B. Volatile Organic Compounds (VOCs):

The boiling point of these compounds fall in the range of 50–100 to 240–260 °C. Some common examples are formaldehyde, toluene, acetone, ethanol, isopropyl alcohol, hexanal, etc.

C. Semi-Volatile Organic Compounds (SVOCs):

The boiling point of these compounds fall in the range of 240–260 to 380–400 °C. Some common examples of such compounds are pesticides such as DDT, chlordane, phthalates, and fire retardants such as PCBs, PBBs, etc.

### 2.3. Removal of VOCs

The effective removal of harmful gases including volatile organic compounds is of significant importance for personal protection from being exposed to such hazardous compounds as well as environmental protection. In this context, the effective and efficient removal of volatile organic compounds from the environment is inevitable and has attracted a great deal of attention from researchers. Commonly used techniques for the removal of VOCs include thermal and catalytic oxidation, adsorption, condensation, bio-filtration, membrane separation, UV-oxidation, catalytic oxidation, and surface modification [44,45,46]. Some of the common methods for the removal of VOCs are given in Table 1. All measurement methods for VOCs are selective or no method is capable of measuring all VOCs. Therefore, researchers are investigating a cost-effective, efficient, and environmentally friendly technique with high sensitivity, selectivity, and specificity. Physical adsorption or chemical adsorption of gas molecules rely on the surface area, pore size (microporous, mesoporous, and macroporous), atomic coordination, and electron density (electron rich or electron deficit) of solid adsorbent. Highly porous materials explored for volatile organic compound adsorption are activated carbons, porous silica, carbon nanotubes, molecular sieves, and various kinds of zeolites where a major mode of gas entrapment is physical adsorption via van der Waals’ forces [47]. Still, there is plenty of room to create specific adsorption sites on the adsorbent by surface the functionalization process [48]. For this purpose, a highly porous solid material with a rigid outfit with appropriate voids sufficient to lodge the organic moieties of a hydrophilic or lipophilic nature is essential. One such material could be metal–organic framework (MOF) or metal–organic framework-derived nanomaterials with profound surface areas. In terms of increasing the surface efficacy or decreasing the dead volume, a metal–organic framework-derived nanostructured material could be a highly desirable porous material for the removal of volatile organic compounds.

## 3. Some Metal–Organic Frameworks:

Some commonly used metal–organic framework are briefly discussed below.

**Figure 1:**
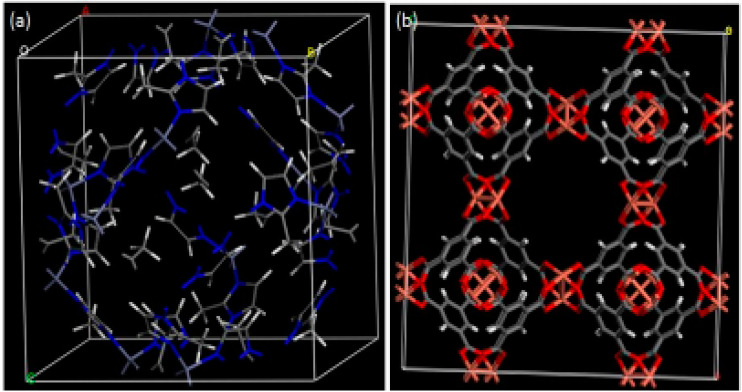
ZIF-8 (**a**) and Cu-BTC (**b**) obtained by modeling (adapted from Boudjema et al., 2019, with permission).

**Figure 2:**
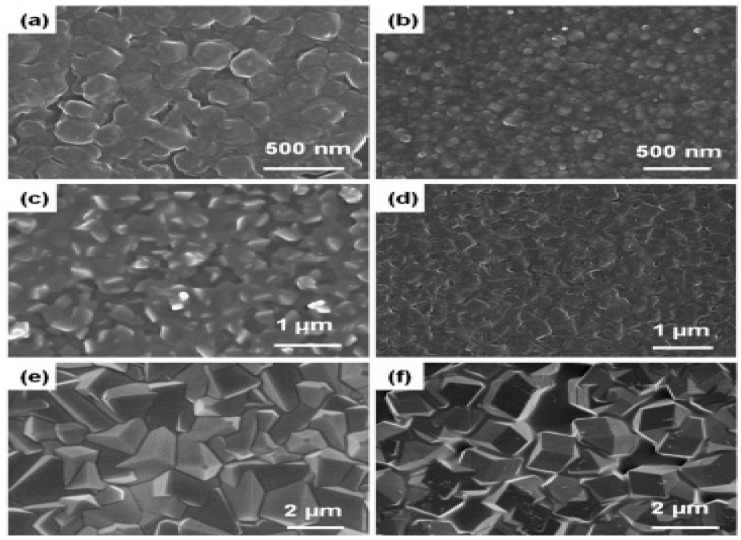
SEM image of ZIF thin films grown on SiO_2_ surface with ten growth cycles: (**a**) ZIF-7, (**b**) ZIF-8, (**c**) ZIF-9, (**d**) ZIF-65-Zn, (**e**) ZIF-67, and (**f**) ZIF-90 (adapted from Tu et al., 2015, with permission).

A ZIF-8 is 2-methylimadizole zinc salt of general chemical formula C_8_H_10_N_4_Zn. The ZIF-8 is Zn(MeIM)_2_. Here, MeIM is 2-methylimidazolate. The ZIF-8 is composed of zinc atom bonded with 2-methylimidazolate ligands with large cavities (11.4 Å) and small pore (3.4 Å) structures (Figure 1a [62]). The different SEM image of different ZIF structures [63] are shown in the Figure 2.
B Cu-BTC is copper benzene-1,3,5-tricarboxylate with a chemical formula of [Cu_3_(btc)_2_] or C_16_H_6_Cu_3_O_12_. Here, btc is 1,3,5-benzenetricarboxylate. It is commercially available. It consists of three distinct cages: one small octahedral cage with a pore window of 2.0 Å and pore radius of 5.2 Å. Another larger cage is the cuboctahedral cage with a pore radius of 6.1 Å connected by a pore aperture of 2.6 Å radius (Figure 1b).

### 3.1. MOF-Derived Nanomaterials for the Removal of VOCs

Nanomaterials are characterized by their high surface area, tunable morphology, and tailorable surface functionality. These days, nanomaterials are gaining superb interest in the field of research as well as in commerce due to their large surface area (aspect ratio); tunable shape, size, and structure; and easiness of fabrication with pronounced magnetic, optical, and electrical properties [64,65,66]. Various tools, techniques, and methods have been devised for the synthesis of desired nanomaterial outfits. The removal of VOCs using active catalyst at low cost, high activity with enhanced surface area is fascinating to researchers. Transition metal oxides, noble metal oxides, and zeolites are commonly used catalysts for the oxidation of VOCs [67]. Contrary to the high-cost noble metal oxide nanomaterials, transition metal oxides are benefitted by their excellent performance at low cost, higher thermal stability, and high natural reserve. Transition metal oxides have variable oxidation states in contrast to non-transition metal oxides, in general. Having such variable oxidation states are better suited for VOCs’ sensing applications. For instance, in the presence of excessive reactive oxygen species (ROS) produced from ionizer, oxidative conversion of Co(II) to Co(III) is possible. The tetrahedral Co(II) having a 3d^7^ configuration changes to less stable tetrahedral 3d^6^ configuration. The reported ligand field stabilization energy (LFSE) for tetrahedral 3d^6^ and 3d^7^ are 0.6Δ_T_ and 1.2Δ_T_, respectively. The Co(III) species becomes favorable to the adsorption of VOCs, especially nitrogen and oxygen donor moieties [68]. Transition metals such as cobalt and manganese have variable oxidation states, which seems very suitable for the redox reaction and oxidation of volatile organic compounds [69]. For higher sensitivity, binary, ternary, or noble metal decorated metal oxides can be used. Furthermore, tuning of the shape, size, composition, surface area, doping level, and fabrication method can enhance the sensitivity performance. Besides these, carbon nanomaterials, nano-biochar, silica nanomaterials, etc. are also commonly used for the removal of VOCs [70,71,72,73]. Some of the commonly used nanomaterials for the removal of VOCs include graphene-based nanomaterials, mesoporous organosilica nanomaterials, carbon nanomaterials, etc. There are various methods for the preparation of nanomaterials including chemical methods, electrochemical methods, electrospinning methods, and so on [74,75,76,77,78,79]. However, the dire need of highly porous nanomaterials may not be addressed by such conventional methods. A new method for the synthesis of highly porous nanomaterials could be by using the metal–organic framework. These days, due to having a high surface area, adjustable aperture, and controllable calcination condition, versatile applications have highly increased an interest on MOFs [80]. Metal clusters as well as organic linkers (ligands), which are the part and parcels of metal–organic frameworks, can be executed for the fabrication of metal or metal oxide nanomaterials as well as carbon nanomaterials, respectively. The synergistic effect of nanoporous carbon wrapped metal or metal oxide nanomaterials can exhibit still more some advantages such as greater stability, dispersion of metal active sites, etc. [81,82]. In this context, the metal–organic framework seems to be a versatile precursor as well as a sacrificial template for the synthesis of porous nanomaterials such as oxides, carbides, chalcogenides, etc. [83]. The synthesis of MOF-derived NMs is benefitted by their controllable compositions and tunable morphologies with affluent porosity endowed, with versatile applications in various fields including sensors and volatile organic compound removal. Zhang et al. reported that MOF-derived (Mn-MIL-100) porous Mn_2_O_3_ cubes displayed sound stability and high activity for the oxidation of carbon mono-oxide over its surface. This method of preparation benefitted from having a high quantity of surface active oxygen, a smaller particle size, and oxygen vacancies along with low temperature reduction behavior [84]. Sun et al. reported the preparation of MnO_x_ by various methods such as thermal decomposition, pyrolysis of MOFs, and co-precipitation. Among them, MOF-derived MnO_x_ exhibited excellent catalytic activity of toluene, which could be attributed to the high surface defects [85]. In many cases, MOFs can be used as templates to develop NMs. The appropriate selection of metal–organic framework precursors with special morphologies under suitable experimental conditions can yield a material of desired morphology. Metal, metal oxides, metal sulfides, and other nanosized materials can be synthesized using a suitable nano-MOFs precursor.

MOFs themselves are a large source of carbon. Metal ions in connection with organic ligands in MOFs can be reduced to metallic composites by the carbothermal reduction process followed by acid etching of metal. The porosity retains its position in the products though the thermal treatment, which may change the pore size distribution within MOFs. MOF-derived porous carbon or carbon–metal porous material can be obtained by direct calcination of MOFs. However, porous carbon material can be prepared by intrusion of a secondary carbon source (e.g., phenolic resin, furfuryl alcohol, ethylenediamine, etc.) either by the wet chemical method or by the vapor phase method followed by carbonization in an inert atmosphere. Nitrogen-doped porous carbon material can be obtained either by the carbonization of nitrogen-rich MOFs (e.g., zeolite imidazolate framework, ZIF-8) or by carbonization of nitrogen-rich organic solution (e.g., dicyanamide) dispersed MOFs. Hetero-element (oxygen, phosphorus, nitrogen, etc.) doping can also be performed by soaking MOFs in organic solution rich in glycophosphine, triarylphosphine (for phosphorus), and dimethylsulphoxide (for sulfur). MOF-derived metal oxides can be obtained by treating MOFs in an excess of air, whereby organic linkers are decomposed, leaving behind metal oxides.

### 3.2. Some Common VOCs and their Removal

Some common VOCs and their removal methods are briefly described below.

Removal of propane and butane:

Propane and butane are commonly used very volatile organic compounds. They are shipped as a liquefied gas and are commonly used for heating and cooking purpose. They are used as common heating fuels in households, heaters to warm garages, and as fuels used for barbecuing, gas grills, and camping lights. These volatile compounds are mostly released from their uses in grills, heaters, and camping lights. These are harmful substances to be inhaled. These gases can be removed by the oxidation process. For instance, propane is converted into water and carbon dioxide via the oxidative reaction. MOF-derived NMs have been used for its removal. Lin et al. synthesized Co_3_O_4_ nanoparticle-assembled micro-rods with Co-BTC (cobalt-1,3,5-benzenetricarboxylic acid) MOFs for the oxidation of propane. Lin et al. proposed the decomposition of propane as the following: two hydrogen atoms are deprotonated from propane and changes to propylene. Then, hydrogen attached to a singly bonded carbon atom is replaced by a hydroxyl group to form allyl alcohol, which is oxidized to ally acid and decomposes to carbon dioxide and water [86].

Removal of methyl chloride:

Methyl chloride is also called chloromethane. It is a colorless, flammable, and toxic gas commonly used in refrigeration and has applications in various industries. It is used as a solvent in petroleum refining, as a methylating and chlorinating agent in organic synthesis, as a propellant in polystyrene foam, and as a herbicide. Methyl chloride is typically present in paint removers, aerosol solvents, and flame retardant chemicals used in fire extinguishers. It is a prominent volatile compound and is prone to photochemical reaction. The inhalation of methyl chloride may cause dizziness and drowsiness depending upon the level of exposure. Kumar et al. synthesized non-activated biochar for the removal of methyl chloride [72]. The MOF-derived highly porous carbon materials could be useful for the removal of volatile organic compounds such as methyl chloride.

Removal of ethanol:

Ethanol is commonly called ethyl alcohol. Ethanol is typically used as in cleaners, sanitizers, laundry detergents, and dishwasher detergents. Ipadeola et al. [87] synthesized Pd/SnO_2_ NPs on MOF-derived carbon for the oxidation of ethanol. Pd/SnO_2_/metal–organic framework-derived carbon (MOFDC) exhibited a superior kinetic parameter in terms of the Tafel slope. This principle is also applicable for direct ethanol fuel cell.

Removal of methanol:

Methanol is commonly called methyl alcohol. Wu et al. synthesized ultrafine Pt NPs and amorphous Ni supported on 3D-mesoporous carbon-derived Cu-MOF for the oxidation of methanol [88]. The carbon matrix lodged for profound dispersion of Pt NPs. The composite catalyst was also found to manifest an outstanding property for the reduction of nitrophenol.

**Figure 3:**
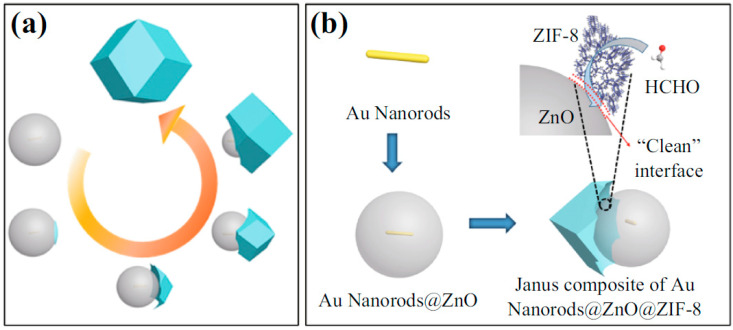
(**a**) Diagrammatic illustration of anisotropic synthesis of AuNRs@ZnO@ZIF-8; (**b**) selective detection of HCHO (adapted from Wang et al., with permission) [90].

**Figure 4:**
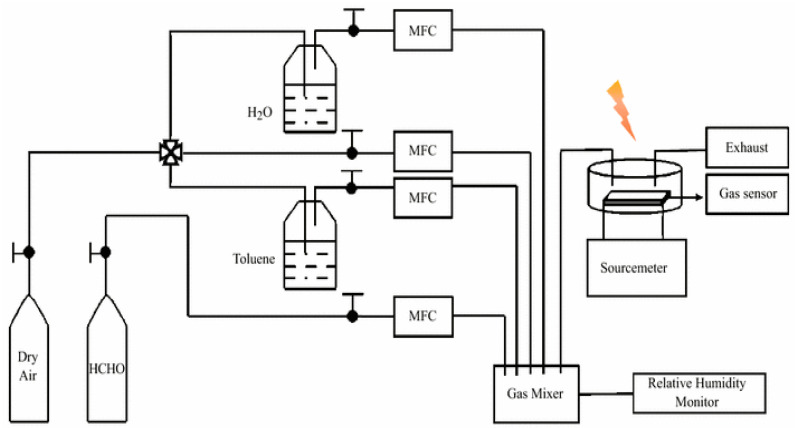
Schematic diagram for the detection of formaldehyde using sensing setup (adapted from Wang et al., with permission [90].

Removal of formaldehyde:

Formaldehyde, also called methanol, is a common volatile organic compound. It is classified as a carcinogenic compound. It is present in molded plastics and coatings such as furniture polish. Nearly 40% formaldehyde is called formalin, which is commonly used in museum specimens. Formaldehyde is used to prepare resins for building materials, and coating for clothing fabrics and paper. It is commonly present in plastics, glues, lacquers, laminate flooring, plywood, fiberboard, particle board, etc. For the detection of formaldehyde, Zhang et al. synthesized ZnO/ZnCo_2_O_4_ microsphere modified by catalytic palladium oxide nanoparticles via an MOF-template route. The microsphere exhibited a fast and higher response, better selectivity, and low detection limit (200 ppb) [89]. Wang et al. developed Janus AuNRs@ZnO@ZIF-8 NPs for the simultaneous detection and removal of formaldehyde (Figure 3a,b). Figure 4 shows the experimental setup for the HCHO detection.

Removal of formic acid:

Formic acid, also called methanoic acid, can be removed by the catalytic decomposition method. Wang et al. developed N-doped C-anchored Pd NPs by the wet chemical method using MOF (ZIF-8) as a precursor. The porous structure of carbon exhibited a high surface area, favoring the decomposition of formic acid [91].

Removal of acetone:

Acetone is also called propanone. It is commonly used as a solvent in laboratory and industrial processes. Everyday sources of exposure to acetone include paints, nail polishes and nail polish remover, furniture polish and wallpaper, and common laboratory environments. Xia et al. synthesized porous Au/ZnO NPs via facial metal–organic framework route for acetone sensing application [92].

Removal of carbon monoxide:

Carbon monoxide is mostly produced from incomplete combustion of any compounds, from common household burning materials to various petrochemicals. Wang et al. developed Co_3_O_4_ nanoparticles by the pyrolysis of cobalt nitrate in the pores of ZIF-8 (Zn(2-methylimidazole)_2_) for the catalytic oxidation of carbon monoxide. Co_3_O_4_ exhibits good cycling and long-term stability. However, one of the disadvantages of using Co_3_O_4_ is that it suffers from deactivation [93]. Catalytic oxidation of carbon monoxide can also be brought about by Mn_2_O_3_. It has outstanding thermal stability. The efficacy of the catalyst is affected by the presence of moisture, operating temperature, etc. [84].

Removal of toluene:

Toluene is also called methyl benzene. Toluene is a good solvent for non-polar compounds. It is sometimes present in paints and coatings. It is an important compound used as an additive in gasoline. For industrial applications, it is used to prepare nylon, plastics, dyes, inks, and paints. Due to its high level of toxicity, these days, toluene-free inks (marker inks) and paints are more often manufactured.

Toluene can be removed by catalytic oxidation or absorption. Zhao et al. synthesized a series of hollow Co_3_O_4_ polyhedron with different sizes by the pyrolysis of ZIF-67. The obtained cobalt-based metal–organic framework owned superior catalytic performance and stability for toluene oxidation [94]. Zhang et al. developed MOF-derived a mesoporous/microporous Mn_2_O_3_ catalyst with a high surface area (141.5 m^2^/g) which exhibited excellent catalytic activity for the oxidation of toluene. They also co-related the higher catalytic efficacy with small crystallite size. The manganese oxide sample obtained from different precursors exhibited different catalytic activities for the oxidation of toluene [95]. Wang et al. developed a series of Zr-based catalysts by the direct decomposition of metal–organic framework UiO-66 in air. In this work, CuCeZr catalyst exhibited an excellent oxidation of carbon monoxide and toluene. For the oxidation test, toluene vapor was carried out by pure argon at the rate of 7 mL/min in a bubbler followed by diluting with 30% O_2_/Ar and another pure Ar. Mass flow controller was used to control the gas flow rate. After 30 min, the conversion of toluene was recorded using an online gas chromatogram with a flame ionization detector (FID) and thermal conductivity detector (TCD) [96]. Zhang et al. prepared highly dispersed silver nanoparticles supported on UiO-66 derivative and studied the effect of silver loading on the structure and performance of catalytic oxidation of toluene. Increasing the silver nanoparticles weight up to 10% (by weight) on UiO-66 caused the collapse of the framework, resulting in the uniform dispersion of silver nanoparticles on the surface. It exhibited an excellent catalytic performance due to higher lattice oxygen and surface silver content. They also unveiled that the catalytic oxidation of toluene led to the formation of benzaldehyde followed by benzoic acid, eventually forming carbon dioxide and water [97].

(1) $$C_{6}H_{5}CH_{3}\;\overset{Catalytic\;oxidation}{\to }\;C_{6}H_{5}CHO\;\overset{Catalytic\;oxidation}{\to }\;C_{6}H_{5}COOH\;\overset{Catalytic\;oxidation}{\to }\;{\mathrm{CO}}_{2}+H_{2}O$$

Zhang et al. developed a series of Pd NPs-loaded UiO-66-NH_2_ using solution impregnation method for the epoxidation of styrene [98].

Removal of xylene:

Xylene vapors are likely to be produced from car tailpipes. *p*-xylene is an isomer of xylene. *p*-xylene is commercially used in the production of polyethylene terephthalate (PET) polymer, beverage bottles, fibers, and films. Separation of p-xylene from its different isomers is of the utmost importance in the petrochemical industry. However, separation is quite difficult by distillation due to their close boiling points (in the range of 138–144 °C). However, separation of p-xylene is possible among its isomers by fractional crystallization and adsorption using zeolites. An MOF containing an extended porous network of cyclodextrin and alkali metal salt has been reported for the separation of xylene regioisomers [99].

Removal of styrene:

Liu et al. developed a highly porous silver-nanoparticle-incorporated MOF for the solar-light-triggered regenerative adsorptive removal of styrene [100]. Herein, silver nanoparticle was incorporated with UiO-66 by the colloidal deposition method. UiO-66 is a zirconium-based metal–organic framework with a high surface area (1180–1240 m^2^/g) and substantial stability [101]. Here, UiO stands for University of Oslo [12]. The particle size of UiO-66 itself is in the range of 100–500 nm. As-developed Ag/UiO-66 samples were highly porous. The incorporation of Ag NPs increased the styrene adsorption capacity compared to the parent UiO-66. Upon exposure to simulated solar radiation, silver nanoparticles induced the conversion of light energy into thermal energy, which triggers the desorption of styrene from the Ag/UiO-66. In this way, Ag/UiO-66 can be regenerated.

Table 2 indicates the MOF-derived nanomaterials applicable in the various VOC gas detection/oxidation. Some nanomaterials fabricated by other methods are also incorporated for a comparative study.

## 4. Challenges and Future Prospects of MOF-NMs

The synthesis of hollow-structured materials with a tunable chemical composition sufficient to sense the volatile organic compounds is still a challenging job. These days, MOFs of versatile applications are being developed. However, the development of MOFs capable of throughput sensing of volatile organic compounds and their selective removal is still a challenging task. The metal–organic framework consists of metal ions or metal oxide clusters in connection with organic moieties creating a profound surface area with significant structural and chemical diversity. In contrast to the traditional adsorbents, such as activated carbon, activated carbon fiber, silicates, and zeolites, metal–organic framework-based nanomaterials (MOF-NMs) have been engineered to tune the adsorption efficacy of volatile organic compounds. Such MOF-NMs are characterized by their tunable pore size and structure, tailorable functionalities, and flexible synthetic methods. A major issue of MOFs is stability, which is largely determined by the structure of metal ions and its nature of bonding with ligands. Weak thermal, chemical, and mechanical stability has limited their use in large-scale applications. The incorporation of nanomaterials has provided some remarkable properties for the MOFs. The incorporation with other functional nanomaterials can greatly improve the sensing performance of MOF [106].

As MOFs act as template for the synthesis of NMs, the design and engineering of an appropriate MOF is a challenging task in the design of MOF-derived NMs. Furthermore, the rampant use of chemicals has absurdly deteriorated the environmental condition as well as health of the researchers, consumers, and allied persons; therefore, the use of green solvents is absolutely necessary. For instance, water and ethanol can be used as green solvents in the synthesis of MOFs.

## 5. Conclusions

With the advent of science and technology, the excessive and rampant use of chemicals cannot be denied. Demand for sophisticated and luxury lifestyles implore the use of high levels of chemicals directly or indirectly in various applications. Many volatile organic compounds are toxic and detrimental to human health and the environment. Such types of volatile organic compounds can be one of the causes of occupational diseases. To be protected from such VOC hazards, there is a significant need to develop a fully satisfactory method for the removal or detoxification of VOCs. Currently, physical, chemical, or biological treatment methods are in action; each method has its own inherent pros and cons, and none of the methods is a panacea. There is an urgent need to devise a promising method for sensing, detoxifying, and removing VOCs. Many cases are not simple, as has been discussed in the literature. A VOC repository may consist of a mixture of various volatile organic compounds rather than some pristine chemicals. Furthermore, a treacherous condition may be found when parts of VOCs are region-selective or stereo-selective. In the chaos of the complex nature and diversity of VOCs, it is hard to find a simple and holistic method for the removal of VOCs at single slot. In this context, to improve the indoor as well as environmental air quality, it is necessary to innovate an efficient, smart, and facile approach for the removal of VOCs. Low-cost, highly efficient, tunable, tailorable, and environmentally friendly nanomaterials with high stability must be assessed by rigorous research. Materials such as carbon-based, metal-oxide-based materials and their variants with promising morphologies, synthesis techniques, and nanoarchitechtonics are needed for the removal of VOCs. In this context, special attention is to be paid to the minimization of VOCs, as well as the removal and detoxification of them.

## Figures and Tables

**Table 1:** Some volatile organic compounds, their sources, and common methods of removal.

SN	VOCs	Sources	Removal Method	Ref
1	Acetaldehyde	Photochemical production	Using carbide-derived carbon	[49]
2	Acetone and toluene	Oxidation of fuels, biomass burning, geochemical process	Using ball-milled biochar	[50,51]
3	Benzene	Traffic and various industries	ZnO NPs coated on zeolite and activated carbons	[52]
4	Chlorinated volatile organic compounds (Cl-VOCs), e.g., monochloromethane, dichloremethane, trichloromethane, etc.	Gas streams	Using activated carbon	[53]
5	Ethyl acetate, isopropanol, acetone		hydoxyapaptites	[54]
6	Formaldehyde and toluene	Photochemical production	Photocatalytic decomposition using SnO_2_ photocatalyst	[55]
7	Methyl tert-butyl ether (MTBE)	Motor fuel additive	Using activated carbon	[44]
8	Toluene	Petrochemical production	Photocatalytic oxidation of toluene to CO_2_ and H_2_O	[56]
9	Toluene		Using Pd supported hierarchical alumina microsphere catalyst	[57]
10	Toluene		Using Fe-MOFs as both an adsorbent and photocatalyst	[58]
11	Toluene		Toluene capture by using imidazolium-based ionic liquids	[59]
12	Toluene		Visible-light-sensitive photocatalytic decomposition of toluene using WO_3_-deposited Pt	[60]
13	Toluene		Solar photocatalytic oxidation of toluene using Co-doped TiO_2_	[61]

**Table 2:** MOF-derived nanomaterials applicable in VOC gas detection or oxidation.

SN		VOCs	Types of Nanomaterials	Efficacy	Preparation Method	Ref.
1		Acetone	Au/ZnO NPs	Gas sensing response of 17.1 ppm^–1^	Calcination of ZIF-8	[92]
3		Benzene	ZnO NPs-coated zeolite and AC	Detection limit of 3 ppb	By coating ZnO NPs and AC on zeolite	[52]
4		Benzene	MnO_2_/ZSM-5 zeolite	Benzene can be removed completely. CO_2_ selectivity reached to 84.7%	Impregnation of metal oxide on ZSM-5	[102]
6		Carbon monoxide	Co_3_O_4_	-	Sacrificial removal of MOFs	[93]
7		Carbon monoxide	Mn-MIL-100-derived Mn_2_O_3_ nonporous	-	Calcination of MOFs at 700 °C.	[84]
9		Ethanol	Pd/SnO_2_ NPs on MOF-derived carbon	-	Microwave-assisted method	[87]
10		Formaldehyde	PdO NPs-decorated ZnO/ZnCo_2_O_4_ microsphere	Detection limit of 0.2 ppm	Prussian-blue-based co-precipitation using MOF (Zn_3_[Co(CN)_6_]_2_)	[89]
11		Formic acid	N-doped C-anchored Pd NPs	Turn over frequency of the catalyst at 30 °C is 1166 h^–1^.	Wet chemical method using ZIF-8	[91]
12		Methanol	Pt NPs and amorphous Ni supported 3D mesoporous C	Diverse selectivity on nitrophenol	Carbonization and chemical etching of Cu-MOF	[88]
13		Naphthalene	CeO_2_	-	Homogeneous precipitation method with urea	[103]
14		Propane Toluene	Mesoporous α-Fe_2_O_3_	-	Wet chemical synthesis	[104]
15		Propane	Co-BTC	-	Hydrothermal method	[86]
16		Styrene	Pd/UiO-66-NH_2_	Highest conversion (87%) of styrene and best selectivity (96.5%) in acetonitrile	Solution impregnation method	[98]
17		Styrene	Ag/UiO-66	-	Colloidal deposition method	[100]
18		Toluene	Hierarchical porous carbon	Adsorption performance of 2290 m^2^/g	Microbial lignocellulose decomposition	[105]
19		Toluene	Mn_2_O_3_	-	Pyrolysis of MOFs containing Mn salts	[95]
20		Toluene	Ag/UiO-66	-	Liquid phase reduction	[97]
21		Toluene	MnOx-CeO_2_-MOF derived from MOF	-	In situ pyrolysis of MOF-74	[85]
22		Toluene	Hollow Co_3_O_4_ polyhedral nanocages	Complete conversion of toluene was observed at 280 °C	Pyrolysis of ZIF-67 MOFs	[94]
23		Xylene isomers	Cyclodextrin-alkali metal salt MOFs MIL-101 (Cr)	The equilibrium capacities of o-xylene, m-xylene and p-xylene are 175, 70, and 64 mg/g, respectively	Wet chemical method	[99]
24		Toluene and CO	CuCeZr700	CuCeZr700 exhibited 100% of CO oxidation at 140 °C and 90% toluene oxidation at 310 °C	Direct decomposition of UiO-66 MOFs in air	[96]

## Abbreviations

BDC	1:4-Benzenedicarboxylate
BTC	1,3,5-benzenetricarboxylic acid
BTEX	Benzene, toluene, ethylbenzene, and xylene
COF	Covalent organic framework
DDT	Dichlorodiphenyl trichloroethane
EPA	Environmental Protection Agency
FID	Flame ionization detector
LFSE	Ligand field stabilization energy
MOF	Metal–organic framework
PAH	Polycyclic aromatic hydrocarbons
PBB	Polybrominated biphenyl
PCB	Polychlorinated biphenyl
PCPs	Porous coordination polymer
PET	Polyethylene terephthalate
PM	Particulate matter
PVC	Polyvinyl chloride
ROS	Reactive oxygen species
SOA	Secondary organic aerosol
SVOC	Semi-volatile organic compounds
TCD	Thermal conductivity detector
UiO	University of Oslo
VOC	Volatile organic compound
VVOC	Very volatile organic compounds
WHO	World Health Organization
ZIF	Zeolite imidazolate framework
ZIF-8	Zn(2-methylimidazole)_2_
